# Suicides, Homicides, Accidents, and Other External Causes of Death among Blacks and Whites in the Southern Community Cohort Study

**DOI:** 10.1371/journal.pone.0114852

**Published:** 2014-12-08

**Authors:** Jennifer S. Sonderman, Heather M. Munro, William J. Blot, Robert E. Tarone, Joseph K. McLaughlin

**Affiliations:** 1 International Epidemiology Institute, Rockville, Maryland, United States of America; 2 Vanderbilt-Ingram Cancer Center, Nashville, Tennessee, United States of America; Medical University of Vienna, Austria

## Abstract

Prior studies of risk factors associated with external causes of death have been limited in the number of covariates investigated and external causes examined. Herein, associations between numerous demographic, lifestyle, and health-related factors and the major causes of external mortality, such as suicide, homicide, and accident, were assessed prospectively among 73,422 black and white participants in the Southern Community Cohort Study (SCCS). Hazard ratios (HR) and 95% confidence intervals (CI) were calculated in multivariate regression analyses using the Cox proportional hazards model. Men compared with women (HR = 2.32; 95% CI: 1.87–2.89), current smokers (HR = 1.74; 95% CI: 1.40–2.17), and unemployed/never employed participants at the time of enrollment (HR = 1.67; 95% CI 1.38–2.02) had increased risk of dying from all external causes, with similarly elevated HRs for suicide, homicide, and accidental death among both blacks and whites. Blacks compared with whites had lower risk of accidental death (HR = 0.46; 95% CI: 0.38–0.57) and suicide (HR = 0.55; 95% CI: 0.31–0.99). Blacks and whites in the SCCS had comparable risks of homicide death (HR = 1.05; 95% CI: 0.63–1.76); however, whites in the SCCS had unusually high homicide rates compared with all whites who were resident in the 12 SCCS states, while black SCCS participants had homicide rates similar to those of all blacks residing in the SCCS states. Depression was the strongest risk factor for suicide, while being married was protective against death from homicide in both races. Being overweight/obese at enrollment was associated with reduced risks in all external causes of death, and the number of comorbid conditions was a risk factor for iatrogenic deaths. Most risk factors identified in earlier studies of external causes of death were confirmed in the SCCS cohort, in spite of the low SES of SCCS participants. Results from other epidemiologic cohorts are needed to confirm the novel findings identified in this study.

## Introduction

External causes of death comprise a heterogeneous collection of events including the three major categories of suicide, homicide, and accidental death. These causes of death represent a significant proportion of potentially preventable mortality in the United States (US). In 2010, suicides and accidents occupied two of the top 10 leading causes of death [1]. Homicide only recently fell from the top 15 causes of death (to 16^th^) for the first time since 1965 [1].

In 2010, US whites had a much higher rate of death due to suicides (14.1 per 100,000) than US blacks (5.1 per 100,000) [1], [2]. The rate of death due to accidents was also higher among whites (42.8 per 100,000) than among blacks (28.7 per 100,000). Conversely, blacks had a much higher death rate due to homicide (18.6 per 100,000) than whites (3.2 per 100,000) [1]. Earlier studies have identified factors aside from race associated with deaths due to suicide, homicide, or accidents, with suicide being studied most but with few studies examining risk factors for all the categories of external causes in one study cohort. Reported or proposed risk factors for external causes include male sex [3], [4], low socioeconomic status (SES) [3], [5], [6], alcohol use [4], [6]–[8], tobacco use [7], [9]–[15], marital status [3], [16], short stature (for homicide) [5], [6], and depression (primarily for suicide) [9], [17], [18]. Racial differences have persisted after adjustment for these factors in the US [19], and it has been suggested that risk factors for suicide among whites, for example, alcohol consumption, may not be a risk factor among blacks [8], [20]. Religiosity has also been suggested to reduce suicide risk among blacks in the US, but the evidence has been inconsistent [4], [16], [21].

The Southern Community Cohort Study (SCCS) is comprised of primarily low-income black and white adults enrolled throughout the Southeastern US. This cohort presents a unique opportunity to examine race- and SES-specific associations between commonly observed risk factors for external causes of death in a population of socioeconomically similar black and white adults. Potential risk factors will be evaluated for the major categories of external cause of death, with information about risk factors obtained at the time of enrollment into the cohort.

## Materials and Methods

The SCCS is a prospective cohort study of approximately 85,000 primarily black and white adults designed to study cancer and other health outcomes in underserved populations in the Southeastern US. From 2002–2009, participants 40–79 years of age were enrolled in-person at one of 71 community health centers (CHCs; 85%) or by randomized general population mailings (GP; 15%) from the following 12 Southeastern states: Alabama; Arkansas; Florida; Georgia; Kentucky; Louisiana; Mississippi; North Carolina; South Carolina; Tennessee; Virginia; and West Virginia. Participants completed a questionnaire at enrollment which gathered information on demographic, lifestyle, personal and family medical history, and other factors [22], [23].

SCCS participants are actively followed through administration of follow-up questionnaires, and passively followed through linkages with state and national registers. All-cause and cause-specific mortality in the SCCS is ascertained through linkage with the National Death Index, currently available through 2011 (http://www.cdc.gov/nchs/ndi.htm). Alive status is ascertained through contact with the participants and linkage with the Social Security Administration's Service for Epidemiologic Research (http://www.ssa.gov/policy/about/epidemiology.html).

To address possible geographic differences in rates of death due to external causes, directly standardized rates of death (2000 US population standard) for the population age 40 years and older were calculated for the 12 SCCS states and the 38 remaining states for the years 2002 through 2010. The age-adjusted rates and corresponding standard errors were calculated using version 8.1.2 of SEER*Stat [24]. The ratio of the death rate in the SCCS states to the death rate in the non-SCCS states was calculated by race and sex for categories of external cause of death, and the approximate 95% confidence interval (CI) for each rate ratio was calculated by transforming the confidence interval for the logarithm of the rate ratio (Panel A of Table 1).

**Table 1:** Rate ratios for external causes of death among the population age 40 and over in the 12 SCCS states, and in the SCCS population, by race and sex.

	Panel A								Panel B							
Cause of Death (ICD-10)	SCCS States								SCCS							
	White Males		White Females		Black Males		Black Females		White Males		White Females		Black Males		Black Females	
	Rate Ratio	95% CI	Rate Ratio	95% CI	Rate Ratio	95% CI	Rate Ratio	95% CI	SMR	95% CI	SMR	95% CI	SMR	95% CI	SMR	95% CI
All external (V01-Y89)	1.24	1.23–1.25	1.25	1.24–1.26	1.14	1.12–1.16	1.09	1.06–1.11	2.39	2.02–2.80	2.24	1.83–2.73	1.53	1.34–1.74	1.47	1.21–1.77
Accident (V01-X59, Y85-Y86)	1.22	1.21–1.23	1.23	1.22–1.24	1.20	1.17–1.22	1.12	1.09–1.15	2.87	2.37–3.45	2.28	1.78–2.88	1.38	1.17–1.63	1.26	0.99–1.59
Homicide (X85-Y09, Y87.1)	1.51	1.47–1.56	1.54	1.48–1.61	0.98	0.94–1.02	0.99	0.92–1.06	5.39	3.08–8.75	3.33	1.33–6.85	2.04	1.55–2.63	2.33	1.44–3.56
Suicide (X60-X84, Y87.0)	1.24	1.23–1.26	1.32	1.29–1.35	1.02	0.97–1.07	0.81	0.73–0.90	0.85	0.49–1.38	1.68	0.94–2.77	1.57	1.00–2.36	2.13	0.92–4.20

**Abbreviations**: CI, Confidence Interval; ICD, International Classification of Diseases; SMR, Standardized Mortality Ratio; SCCS, Southern Community Cohort Study

Panel A: Ratio of the directly standardized rate in the 12 SCCS states (Alabama, Arkansas, Florida, Georgia, Kentucky, Louisiana, Mississippi, North Carolina, South Carolina, Tennessee, Virginia, and West Virginia) to the directly standardized rate in the remaining 38 states.

Panel B: Standardized mortality ratio of SCCS population relative to mortality rates in the 12 SCCS states.

Standardized Mortality Ratios (SMRs), the ratio of observed to expected numbers of deaths, and corresponding 95% CIs were calculated to determine whether the external cause mortality experience of SCCS participants differed from that in the general population in the 12 SCCS states (Panel B of Table1). Observed numbers of deaths were defined according to the International Classification for Disease (ICD) 10^th^ Revision: all external causes (V01-Y89); accidents (V01-X59, Y85-Y86); suicides (X60-X84, Y87.0); and homicides (X85-Y09, Y87.1). Expected numbers of deaths were computed based on age-, calendar year-, race-, and sex-specific rates in the general population of the 12 southeastern SCCS states. Person-years of follow-up began on the participant's enrollment date and continued through the date of death or December 31, 2011, whichever occurred first. Confidence intervals were calculated based on the Poisson distribution for the number of deaths in each external cause of death category.

Cox proportional hazards models were used to estimate hazard ratios (HRs) and 95% CIs of mortality from external causes of death as an underlying or contributing cause of death among SCCS participants after their entry into the cohort. Covariates used in the models included race (black versus white); sex (male versus female); enrollment source (CHC versus GP); alcohol use (continuous number of drinks per day); self-reported doctor diagnosis of or treatment for depression (yes versus no); smoking (current, former versus never); income ($15,000–$24,999, $25,000–$49,999, ≥50,000 versus <$15,000); education (did not complete high school, some college or greater versus completed high school/GED or vocational/technical/business training); marital status (divorced/separated, widowed, single versus married); employment status (unemployed/never employed versus currently employed); body mass index (BMI) (low, BMI<18.5 kg/m^2^; overweight, 25.0 kg/m^2^≤BMI<30.0 kg/m^2^; obese, BMI≥30.0 kg/m^2^; versus normal, 18.5 kg/m^2^≤BMI<25.0 kg/m^2^); height (continuous in centimeters); a comorbidity index (continuous); and the self-reported degree to which faith is a source of comfort or strength (not very much, somewhat, quite a bit, versus a great deal). The comorbidity index was calculated for each cohort member based on diseases reported on the baseline questionnaire. This index was based on the Charlson index [25], with modifications to account for chronic disease information collected using the SCCS enrollment questionnaire. A score of 1 was assigned to each of the following diseases: heart attack or coronary artery bypass surgery; stroke; chronic obstructive pulmonary disease, asthma, or tuberculosis; hepatitis; ulcer; diabetes; Parkinson's disease; high cholesterol or hypertension; and autoimmune disease (i.e., arthritis, Crohn's disease, multiple sclerosis, or lupus). A score of 2 was assigned to cancer (other than non-melanoma skin cancer), and a score of 3 was assigned to HIV-AIDS (versus 6 in the Charlson index, a lower value because of the current availability of effective HIV treatments). The comorbidity index for each participant was the sum of the scores for diseases reported by the person on the baseline questionnaire. Age was used as the time-scale for all models, starting at the age at enrollment and ending at the age of death (for deceased participants), age lost to follow-up, or age as of December 31, 2011 (for censored participants). The proportional hazards assumption was checked by use of time-varying covariates [26].

### Ethics Statement

The SCCS is approved by the Vanderbilt University Institutional Review Board Heath Sciences Committee #1. All SCCS participants provided written informed consent at study enrollment.

## Results

Table 1 (Panel A) presents geographic comparisons of mortality rates for external causes of death for the US population age 40 and older by race and sex. White residents of the 12 SCCS states had significantly higher rates for all external causes of death, including accident, homicide, and suicide, than did white residents of the remaining 38 U.S. states. Black residents of the 12 SCCS states had significantly higher rates for deaths due to accidents, but their death rates from homicide were similar to those of blacks in the remaining 38 states. The suicide rates for black women in the 12 SCCS states were significantly lower than those for black women in the remaining 38 states. Panel B of Table 1 shows that relative to residents in the 12 SCCS states, SCCS participants had higher SMRs for most categories in the table, with the exception of a moderately lower suicide rate among white SCCS males.

After excluding 4,129 participants reporting a race other than white or black and 7,194 participants missing covariate information, 73,422 SCCS participants were included in the internal cohort analysis. Table 2 compares the distribution by sex and race for demographic, lifestyle and health-related variables that are potentially associated with external causes of death. White SCCS participants tended to have higher incomes and slightly higher educational attainment and were much more likely to report depression and to be married than black participants. Blacks were much more likely to report religion, faith or God as a source of comfort, and were slightly greater consumers of alcoholic drinks than white participants.

**Table 2:** Distribution of demographic, lifestyle, and health-related variables for Southern Community Cohort Study participants by race and sex.

	Black Females N = 29484		Black Males N = 21124		White Females N = 14063		White Males N = 8751	
Variable Category	N	%	N	%	N	%	N	%
Education									
<High School		8845	30.0	7105	33.6	3460	24.6	2024	23.1
High School/GED/Vocational		11446	38.8	8677	41.1	5480	39.0	3108	35.5
>High School		9193	31.2	5342	25.3	5123	36.4	3619	41.4
Household Income									
<$15,000		17546	59.5	12782	60.5	6994	49.7	3819	43.6
$15,000–24,999		6828	23.2	4539	21.5	2764	19.7	1526	17.4
$25,000–49,999		3706	12.6	2603	12.3	2290	16.3	1481	16.9
≥$50,000		1404	4.8	1200	5.7	2015	14.3	1925	22.0
Marital status									
Married		7892	26.8	6623	31.4	6327	45.0	4561	52.1
Divorced/Separated		10273	34.8	7144	33.8	4792	34.1	2620	29.9
Widowed		4117	14.0	769	3.6	1856	13.2	315	3.6
Single		7202	24.4	6588	31.2	1088	7.7	1255	14.3
Employment Status									
Currently Employed		11798	40.0	8049	38.1	5347	38.0	3506	40.1
Unemployed/Never Employed		17686	60.0	13075	61.9	8716	62.0	5245	59.9
Smoking status									
Current		9705	32.9	12150	57.5	5107	36.3	3717	42.5
Former		5767	19.6	4328	20.5	3567	25.4	2868	32.8
Never		14012	47.5	4646	22.0	5389	38.3	2166	24.8
Body Mass Index (kg/m^2^)									
Low (<18.5)		314	1.1	275	1.3	255	1.8	80	0.9
Normal (18.5–<25.0)		4614	15.6	7430	35.2	3482	24.8	2456	28.1
Overweight (25.0–<30.0)		7478	25.4	7410	35.1	3726	26.5	3221	36.8
Obese (≥30.0)		17078	57.9	6009	28.4	6600	46.9	2994	34.2
Depression^a^									
Yes		6753	22.9	2673	12.7	6273	44.6	2303	26.3
Enrollment Source									
CHC		27536	93.4	19809	93.8	11793	83.9	6347	72.5
GP		1948	6.6	1315	6.2	2270	16.1	2404	27.5
Faith comfort^b^									
Not very much		214	0.7	422	2.0	574	4.1	993	11.3
Somewhat		1183	4.0	1724	8.2	1913	13.6	1729	19.8
Quite a bit		4462	15.1	4818	22.8	3135	22.3	2263	25.9
A great deal		23625	80.1	14160	67.0	8441	60.0	3766	43.0
Cause of Death (ICD-10)									
All external (V01-Y89)		151	0.5	275	1.3	109	0.8	160	1.8
Accident (V01-X59, Y85-Y86)		87	0.3	166	0.8	72	0.5	116	1.3
Homicide (X85-Y09, Y87.1)		20	0.1	56	0.3	6	0.04	16	0.2
Suicide (X60-X84, Y87.0)		8	0.03	21	0.1	14	0.1	14	0.2
Other external		37	0.1	33	0.2	19	0.1	15	0.2
		Mean	SD	Mean	SD	Mean	SD	Mean	SD
Height in cm		164.1	7.2	177.9	8.1	163.3	6.9	178.0	7.2
Alcoholic drinks per day		0.8	2.9	2.8	5.6	0.4	2.0	1.9	4.9
Comorbidity index		2.0	1.5	1.6	1.4	2.2	1.6	1.9	1.5

**Abbreviations**: CHC, Community Health Center; GP, General Population; ICD, International Classification of Diseases; SD, Standard deviation.

a Response to question, “Has a doctor ever told you that you have depression or have you been treated for depression?”

b Response to question, “How much is religion, faith, or God a source of strength and comfort to you?”

In total, there were 695 deaths from all external causes among SCCS participants included in the analysis: 441 deaths (64%) from accidental causes, 98 deaths (14%) from homicide, 57 suicide deaths (8%), and 104 deaths (15%) from other external causes, of which 80 (77%) were the result of complications of medical and surgical care (iatrogenic causes; ICD-10 codes Y40-Y84 and Y88). There was evidence of a lack of fit of the proportional hazards model for accidental deaths (*P*<0.05), but no evidence of lack of fit for homicides, suicides, or deaths from other external causes (*P*>0.2). To assess the impact of the lack of model fit for accidental deaths, logistic regression was used to analyze accidental deaths stratified on year of enrollment. The logistic regression yielded relative risk estimates and CIs very similar to those obtained using the proportional hazards analysis, and thus the results of the proportional hazards analyses will be presented for all categories of external death.

Relative risks for dying from external causes, estimated as hazard ratios, are reported in Table 3 for SCCS participants. For all external causes, men, smokers, and unemployed/never employed participants had much higher risks of dying in both races. Risks of dying were lower in blacks, in participants in the highest two income levels, and in obese participants. Risk of death from all external causes was increased in participants reporting depression, and increased with increasing level of alcohol consumption and with reported number of chronic health conditions. The death rate from all external causes was significantly increased in participants recruited from CHCs, even after adjustment for demographic and lifestyle variables.

**Table 3:** Hazard ratios for risk of death by specific causes corresponding to demographic, health-related, and lifestyle variables in the Southern Community Cohort Study.

	All External Causes ICD-10 V01-Y89		Accident ICD-10 V01-X59, Y85-Y86		Homicide ICD-10 X85-Y09, Y87.1		Suicide ICD-10 X60-X84, Y87.0	
Variable Category	HR^a^	95% CI	HR^a^	95% CI	HR^a^	95% CI	HR^a^	95% CI
Race								
Black	0.58	0.49–0.69	0.46	0.38–0.57	1.05	0.63–1.76	0.55	0.31–0.99
White	1.0		1.0		1.0		1.0	
Sex								
Male	2.32	1.87–2.89	2.28	1.73–3.01	3.24	1.77–5.92	3.02	1.40–6.49
Female	1.0		1.0		1.0		1.0	
Education								
<High School	0.93	0.78–1.11	1.00	0.81–1.25	1.14	0.72–1.79	0.23	0.09–0.60
High School/GED/Vocational	1.0		1.0		1.0		1.0	
>High School	1.00	0.82–1.21	0.98	0.76–1.25	0.93	0.54–1.62	1.20	0.68–2.14
Household Income								
<$15,000	1.0		1.0		1.0		1.0	
$15,000–24,999	0.96	0.78–1.17	0.99	0.77–1.26	0.55	0.29–1.06	1.20	0.62–2.36
$25,000–49,999	0.79	0.59–1.05	0.79	0.55–1.13	0.76	0.32–1.81	1.34	0.59–3.06
≥$50,000	0.67	0.43–1.04	0.45	0.23–0.85	1.99	0.68–5.81	0.89	0.25–3.15
Marital status								
Married	1.0		1.0		1.0		1.0	
Divorced/Separated	1.19	0.98–1.45	1.11	0.87–1.42	2.44	1.26–4.70	0.98	0.50–1.90
Widowed	1.35	1.00–1.84	1.28	0.88–1.87	2.30	0.79–6.72	0.95	0.26–3.39
Single	1.05	0.83–1.31	0.99	0.75–1.32	2.10	1.06–4.18	1.02	0.47–2.20
Employment Status								
Currently Employed	1.0		1.0		1.0		1.0	
Unemployed/Never Employed	1.67	1.38–2.02	1.56	1.23–1.98	1.78	1.08–2.95	1.51	0.81–2.84
Smoking Status								
Current	1.74	1.40–2.17	1.78	1.35–2.35	2.39	1.23–4.67	1.88	0.88–4.00
Former	1.31	1.02–1.69	1.29	0.94–1.78	1.51	0.65–3.51	1.52	0.64–3.63
Never	1.0		1.0		1.0		1.0	
Body Mass Index (kg/m^2^)								
Low (<18.5)	0.80	0.42–1.50	0.75	0.33–1.70	0.50	0.07–3.61	0.98	0.13–7.34
Normal (18.5–<25.0)	1.0		1.0		1.0		1.0	
Overweight (25.0–<30.0)	0.77	0.65–0.93	0.85	0.68–1.07	0.52	0.31–0.86	0.63	0.33–1.21
Obese (≥30.0)	0.62	0.51–0.75	0.63	0.49–0.81	0.62	0.37–1.05	0.52	0.27–1.03
Depression^b^								
No	1.0		1.0		1.0		1.0	
Yes	1.30	1.09–1.55	1.21	0.97–1.51	1.47	0.91–2.38	3.05	1.70–5.48
Enrollment Source								
CHC	1.49	1.04–2.15	1.70	1.05–2.74	UD^c^	UD^c^	1.16	0.44–3.09
GP	1.0		1.0				1.0	
Faith comfort^d^								
Not very much	1.13	0.76–1.68	1.19	0.75–1.90	0.35	0.05–2.58	2.37	0.87–6.45
Somewhat	1.16	0.91–1.48	1.00	0.73–1.37	1.80	1.04–3.11	1.39	0.62–3.12
Quite a bit	1.15	0.96–1.37	1.13	0.90–1.41	0.98	0.59–1.62	1.40	0.75–2.65
A great deal	1.0		1.0		1.0		1.0	
Height (cm)	1.00	0.99–1.01	1.00	0.99–1.02	1.00	0.97–1.02	0.98	0.95–1.02
Alcoholic Drinks Per Day	1.02	1.01–1.04	1.03	1.02–1.05	1.03	1.00–1.05	1.01	0.96–1.06
Comorbidity Index	1.10	1.05–1.16	1.12	1.05–1.19	0.98	0.84–1.14	1.07	0.90–1.28

**Abbreviations:** CI, Confidence Interval; CHC, Community Health Center; GP, General Population; HR, Hazard Ratio; ICD, International Classification of Diseases.

a Hazard Ratios adjusted for age (as time-scale), race, sex, enrollment source, alcohol use, depression, smoking, income, education, marital status, employment status, BMI, height, comorbidity index, faith comfort.

b Response to question, “Has a doctor ever told you that you have depression or have you been treated for depression?”

c Hazard ratio is undefined – no deaths from homicide in the general population enrollees.

d Response to question, “How much is religion, faith, or God a source of strength and comfort to you?”

Accidents accounted for 64% of deaths due to external causes among SCCS participants, and thus the relative risk estimates for accidental deaths were similar to those for all external causes (Table 3). Men had a much higher risk of accidental death than women, and blacks had a much lower risk of accidental death than whites. Current cigarette smokers had almost a two-fold increased risk of accidental death compared with never smokers. The risk of accidental death increased with increasing level of alcohol consumption and with reported number of chronic conditions, and decreased with increasing income level. The accidental death rate was significantly increased in CHC enrollees compared with GP enrollees.

The rate of death from homicide was much higher in male SCCS participants, and was similar in whites and blacks (Table 3). Death rates from homicide were increased 2.4-fold in current cigarette smokers compared with never smokers, and increased with increasing level of alcohol consumption, although the association was not statistically significant. Being married was a strong protective factor for death from homicide. No deaths from homicide were observed among GP SCCS participants (*P*<0.0001 using an exact unadjusted test for 2×2 tables stratified by sex and race to compare the proportions of death from homicide for CHC enrollees with those for GP enrollees).

Suicide rates were much higher in male SCCS participants than in female participants and were much lower in blacks than in whites (Table 3). The strongest predictor of suicide was self-reported physician-diagnosed depression, and suicide risk was increased almost two-fold among current cigarette smokers compared with never smokers. SCCS participants with the lowest educational attainment had a significantly decreased suicide risk, and there was no difference in suicide rates for participants recruited in CHCs compared with the GP recruits. There was a non-significant inverse relation between the extent of comfort received from religion and risk of suicide, which becomes statistically significant when restricted to CHC enrollees (HR = 3.28; 95% CI, 1.20–8.94 for “not very much” comfort versus “a great deal” of comfort).

Overweight and obese participants had reduced risks, and unemployed/never employed participants had increased risks, of dying from all external causes, including accidents, homicides and suicides.

The comorbidity index was significantly associated with death rates from external causes other than suicide, homicide, or accident; risk of death increased with an increasing comorbidity index. A significant hazard ratio for the comorbidity index was observed only for the subset of these deaths resulting from iatrogenic causes (HR = 1.26; 95% CI, 1.10–1.46). There were no significant differences in risk of death from iatrogenic causes by race, sex, or by enrollment source.

## Discussion

Blacks in the SCCS had 50% lower risks of suicides and accidental deaths compared with whites, consistent with expectation based on national mortality rates [1]. The absence in our cohort of an increased risk of homicide deaths among blacks compared with whites was unexpected in view of previous studies [3], [6] and national statistics [1]. Examination of Table 1 indicates that the absence of an increased relative risk is explained by an unusually high homicide rate among white participants in the SCCS.

Consistent with most published studies [3], [4], being male was a very strong risk factor for dying from external causes in SCCS participants. Being male was a risk factor for suicide, homicide, and accidental death, and was associated with the highest observed estimated relative risk for homicide and accidental death. For homicide and accidental deaths the male excess is not surprising, and may be explained by greater exposure to occupational hazards, increased participation in physical/dangerous leisure-time activities, greater tendency to adopt dangerous lifestyle practices (e.g., alcohol consumption and cigarette smoking), and a generally more aggressive nature [27]. Gender differences in suicide rates may be more dependent upon cultural practices, as evidenced by the absence of a male excess in suicide in a recent large study in China [28]. The Chinese study found a highly significant 2-fold higher rate of death among men compared with women for deaths from external causes other than suicide (over 85% of such deaths were accidental deaths).

Current cigarette smoking was associated with about a two-fold relative risk for suicide in SCCS participants. Perhaps no risk factor for suicide has been examined more extensively than cigarette smoking, and a direct relation between smoking and suicide has been observed in most studies [9]–[14]. The explanation for the association between cigarette smoking and suicide risk is unknown; it has been suggested that cigarette smoking may be a marker for stress or psychiatric problems, and residual confounding with alcohol or drug abuse is possible, but a direct role of cigarette smoking on brain levels of serotonin or monoamine oxidases has not been ruled out [10], [14], [29].

Cigarette smoking was also strongly associated with death from homicide and accidental death in our study. A recent follow-up study based on the National Health Interview Study (enrollees interviewed in 1990–1991 and followed for mortality through 1995) reported relative risk estimates consistent with increased risk in heavy smokers for suicide, homicide, and accidental death [12]. A meta-analysis of cohort studies evaluating all injury causes of death (including suicide, homicide, and accidental death) reported a significant association for cigarette smoking [30], and a meta-analysis of randomized trials of smoking cessation reported non-significant decreases in suicide and accidental death (but not homicide death) associated with smoking cessation [31]. Other studies have found no association between cigarette smoking and external causes of death other than suicide [9], [28]. Cigarette smoking could contribute directly to some accidental deaths (e.g., vehicular accidents and fire-related deaths), but as was the case with suicides, cigarette smoking may also be a marker for stress and/or disorders that are associated with increased risk of homicide death or accidental death. Residual confounding (e.g., with alcohol consumption or depression) cannot be ruled out.

Alcohol consumption was significantly associated only with accidental death in the SCCS; the absence of statistical significance for homicides likely reflects the much smaller number of these deaths. Although not studied extensively in epidemiologic studies of homicide or accidental death, a direct association with alcohol consumption is not unexpected [6], [7]. Two case-control studies based on the National Mortality Followback Survey suggested that alcohol consumption might be associated with increased suicide risk among whites, but not among blacks [8], [20]. These mortality case-control studies are based on information obtained from interviews of surrogate respondents for people who have died, and thus the quality of the risk factor information is questionable [32]. The study by Castle et al. [20] is particularly problematic, because controls for suicides were selected from accidental deaths, and some factors, for example, depression and alcohol consumption, may increase the risk of both suicide and accidental death. There was no evidence of a difference between whites and blacks in risk factors for suicide in the SCCS. In particular, no strong relation between alcohol consumption and suicide risk was observed for either blacks (HR = 1.02; 95% CI, 0.97–1.09) or whites (HR = 0.97; 95% CI, 0.87–1.09).

Depression was the strongest risk factor for suicide in the SCCS, a finding that was not unexpected [9], [17], [29]. A possible role of religious belief, or the increased social integration among the religious [33], in reducing the risk of suicide was observed, with a significant association observed among the lower SES CHC participants. Depression was also significantly associated with accidental death in the SCCS; the increased risk of accidental death in people with psychiatric diagnoses, including depression, has been noted previously [17], [18]. The observation of low suicide risk in participants with the least education was unexpected, and requires confirmation.

Unemployed and never employed participants had a significantly increased risk of external mortality compared with currently employed participants at SCCS enrollment. This is consistent with findings of excess homicide [3], [19], suicide [3], [8], [19], [28], and other injury deaths [3], [19] observed among unemployed individuals in previous studies. Unemployment may affect all external causes of death as a surrogate for unmeasured mental illness [29], [33], while recent job loss, which we were unable to examine in the present study, and unemployment are stressors influencing suicide risk [2], [33], [34], [35].

The finding of lower risk of all external deaths in our study, including accidents, homicide and suicides in overweight and obese individuals in the SCCS was a surprise. It is possible that heavier individuals are less likely to engage in certain types of risky activities, and added girth may provide protection in certain types of accidents, but these intriguing findings require confirmation in future studies.

Two studies investigating SES and homicide risk in men have found short stature (evaluated as a surrogate measure of socioeconomic disadvantage early in life) to be a risk factor for homicide [5], [6]. There was no evidence in the present study to support this finding. The only SES association observed in the current study was an increased risk of death from accidents among individuals with low income; low educational attainment was not significantly associated with increased risk of homicide.

Being married was a strongly protective factor against homicide deaths among SCCS participants. Marital status was not associated with any other external cause of death in the SCCS. However, in a nation-wide follow-up study based on the National Health Interview Study (enrollees interviewed in 1987–1994 and followed for death through 1995) in the United States, being married was protective against deaths from suicide, homicide, and accident [3].

The comorbidity index was significantly associated with accidental death and with iatrogenic causes of death. The latter association is plausible, in view of the likely increased need for medical care, including increased use of pharmaceuticals, hospital admissions, and/or surgeries, associated with a greater number of chronic diseases. The association with accidental deaths may reflect increased frailty associated with multiple chronic diseases, or may also possibly be explained by increased use of drugs to treat the greater number of chronic diseases.

CHC enrollment was significantly associated with increased homicide and accident deaths compared with GP enrollment, even after adjustment for demographic, lifestyle, and health-related covariates. This may reflect a contribution of factors not covered by the enrollment questionnaire or possibly the influence of community effects. Such effects related to residential environment have been observed in a study of homicide deaths [19] based on the National Health Interview Study.

The availability of information from SCCS participants at study enrollment about a variety of demographic, lifestyle, and health-related variables is a strength of the current study. Few studies have evaluated the risk of death for the major external cause of death categories in a prospective cohort study setting, or had the ability to investigate the number and variety of potential risk factors included in the present analyses, though these risk factors were assessed only at study entry. The SCCS population is not representative of the US as a whole, particularly with respect to SES. However, even in this low SES population, most risk factors associated with external causes of death in other populations were also identified as risk factors in the SCCS. Novel findings, such as the inverse associations of obesity with suicides, homicides, and accidents; the similarity of homicide rates among low SES blacks and whites; and the relation between iatrogenic deaths and comorbidities need confirmation in other study populations.
